# Sex-Specific Trends in Temporary Mechanical Circulatory Support Use as Bridge to Orthotopic Heart Transplant

**DOI:** 10.1016/j.jacadv.2026.102793

**Published:** 2026-06-17

**Authors:** Nicole Cyrille-Superville, Priyesh A. Patel, Brian N. White, Joseph D. Mishkin, Snehal R. Patel, Rachel Garcia, Heather Rose, Susan Bernardo, Lauren Harmon, Shuktika Nandkeolyar, Sanjeev K. Gulati, Ruobing Xue, William Saunders, Onyedika Ilonze, Amar Doshi, Theodore A. Frank, Adam D. Devore

**Affiliations:** a Sanger Heart and Vascular Institute, Atrium Health, Charlotte, North Carolina, USA; b Wake Forest University School of Medicine, Winston-Salem, North Carolina, USA; c Department of Medicine, Division of Cardiology Northwell Health, New York, New York, USA; d Krannert Cardiovascular Research Center, Indiana University School of Medicine, Indianapolis, Indiana, USA; e Department of Medicine, Duke University School of Medicine, Durham, North Carolina, USA

**Keywords:** device, heart, mechanical, sex-specific, transplant, trend

## Abstract

**Background:**

Following the United Network for Organ Sharing (UNOS) allocation change, patients listed for orthotopic heart transplant are increasingly bridged with temporary mechanical circulatory support (tMCS); however sex-specific data are limited.

**Objectives:**

The authors sought to examine sex-specific trends in tMCS utilization before and after the allocation change.

**Methods:**

We retrospectively analyzed UNOS data from October 2015 to June 2023, comparing baseline characteristics, tMCS use, and outcomes between sexes across allocation eras. Multivariable regression was used to identify predictors of tMCS utilization, waitlist, and post-transplant mortality.

**Results:**

In the postallocation period, male sex and blood type O were independently associated with tMCS use. Women were more likely bridged with intra-aortic balloon pump (IABP) (67% vs 61% *P* < 0.001) with shorter waitlist times (13 vs 16 days; *P* < 0.001). Extracorporeal membrane oxygenation (ECMO) utilization increased for both men (1.1% to 10%) and women (0.5% to 13%). Waitlist death decreased for both sexes; however, women experienced worse 1-year post-transplant survival (*P* = 0.012). Compared with IABP, nondischargeable/percutaneous left ventricular assist devices (subdistribution HR: 2.2; 95% CI: 1.69-2.88; *P* < 0.001) and ECMO (subdistribution HR: 5.29; 95% CI: 3.98-7.07; *P* < 0.001) were associated with higher waitlist death. ECMO was associated with increased risk of post-transplant mortality (*P* < 0.001).

**Conclusions:**

Post allocation change, women remain less likely to be listed on tMCS and more often bridged with IABP. Although waitlist outcomes have improved independent of sex, women experience worse 1-year post-transplant survival. Device type was the strongest predictor of poor outcomes.

In 2018, the United Network for Organ Sharing (UNOS) allocation system was changed to prioritize patients listed with the greatest clinical urgency for orthotopic heart transplant (OHT), whereby the previous single highest urgency status (1A) was broken down into 3 separately ranked statuses in descending order of illness (new status 1, 2, and 3). Patients in cardiogenic shock supported with extracorporeal membrane oxygenation (ECMO) or other nondischargeable biventricular mechanical circulatory support (MCS) are assigned to the highest urgency status and those with lesser degrees of support are distributed into a descending rank order of priority.^1^^,^^2^ Since the allocation change, there has been a significant increase in patients on temporary MCS (tMCS) listed for OHT, with a simultaneous decrease in patients bridged with durable left ventricular assist devices (LVADs).^3^

Prior studies have highlighted sex disparities among patients receiving durable LVAD, with women on LVAD support less likely to receive OHT and more likely to experience waitlist removal and waitlist death.^4^^,^^5^ Moreover, in cardiogenic shock women have been shown less likely to receive acute tMCS compared with their male counterparts.^6^^,^^7^ We therefore sought to investigate the UNOS registry for sex specific trends in the use and outcomes of tMCS among patients listed for OHT, before and after the UNOS allocation change.

## Methods

A retrospective analysis of the combined UNOS thoracic database was performed. This publicly available data contain no patient identifiers and as such was deemed exempt from Institutional Review Board review. The database contains information on all waitlist registrations and OHT recipients listed or performed in the United States and reported to the Organ Procurement and Transplantation Record since October 1, 1987. The database was reviewed for all adults (aged ≥18 years) listed for OHT between October 1, 2015, and June 28, 2023. Patients with a history of previous OHT and those listed for multiorgan transplants were excluded. Patients listed on intra-aortic balloon pump (IABP), nondischargeable/percutaneous LVADs, total artificial heart (TAH), and venoarterial ECMO for OHT were included in the analysis. Unspecified device configurations, those less frequently used to bridge patients to OHT and durable devices such as nonspecified ECMO, venovenous ECMO, right ventricular assist device, dischargeable LVADs, Heartmate II, Heartware HVAD, Heartmate VE, Heartsaver VAD, Heartmate IP, Heartmate XVE were excluded. The majority of patients included were on tMCS at the time of initial listing though some patients were placed on tMCS while on the waitlist (Supplemental Table 1). Data were analyzed from the time a patient was placed on tMCS and we captured the first device in the patient’s implant data sequence. Era 1 was defined as preallocation change (October 1, 2015−October 17, 2018), and era 2 was defined as postallocation change (October 18, 2018−June 28, 2023). Patients who were listed in the old allocation system but transplanted in the new allocation system were excluded from the analysis. The baseline characteristics and outcomes of patients listed on tMCS in eras 1 and 2 were compared. The model for transplantation rate was fit on the full cohort, which included any patient on the OHT waitlist who satisfied the inclusion/exclusion criteria discussed above. In contrast, the models for post-transplant survival were fit on subsets of the data that included only those patients who received OHT.

### Statistical analysis

The statistical analysis was performed on processed thoracic national Standard Transplant and Research files obtained from UNOS. Descriptive statistics and tests of association were calculated for relevant patient characteristics, stratified by UNOS allocation period and sex. Continuous variables are presented as median (IQR) and compared between groups using the Wilcoxon rank-sum test, a nonparametric approach that does not assume normality. Categorical variables are presented as count (%) and compared using Pearson chi-squared test or Fisher exact test when expected cell counts were below 5. A directed acyclic graph was generated, using DAGitty software, to guide selection of covariates (Supplemental Figure 1). We adjusted models for conventional sociodemographic covariates such as age, sex, ethnicity in addition to clinical covariates based on prior literature. These included baseline comorbidities, surrogate markers of illness severity, factors that impact waitlist time and post-transplantation outcomes such as diabetes status, presence of a defibrillator, renal function, cardiac output (CO), cardiac index (CI), blood type, body mass index (BMI)/body surface area (BSA), panel reactive antibody (PRA) where applicable. Details on center-level and provider-level practices, geographic distance for organ offers/organ availability, and donor characteristics are not fully assessed in UNOS and were therefore not incorporated. Blood type A served as the reference category for blood group and IABP as the reference category for device type within models.

Transplant rates were modeled using multivariable logistic regression with a sex-by-allocation-period interaction term, adjusted for age at listing, ethnicity, BMI, diabetes status, implantable defibrillator status, blood group, and MCS device type. Model fit was assessed using the Hosmer-Lemeshow goodness-of-fit test. MCS device use was modeled similarly, with additional adjustment for serum creatinine, PRA, BSA, CO, and CI.

Predictors of waitlist mortality were evaluated using a Fine-Gray subdistribution hazards model to account for the competing risk of heart transplantation. In this analysis, waitlist death was treated as the event of interest, heart transplantation as the competing event, and patients who experienced neither event were censored at last follow-up. Results are reported as subdistribution HRs (sHRs) with 95% CIs. Covariates included sex, age, ethnicity, BMI, diabetes status, blood group, device type at listing, and allocation era based on clinical relevance and prior literature. A sex-by-allocation-period interaction term was also included. Patients with missing data on any covariate were excluded from the model.

Post-transplant survival was analyzed using Cox proportional hazards regression with follow-up administratively censored at 1 year. The model included a sex-by-allocation-period interaction term and was adjusted for demographic and clinical covariates. The proportional hazards assumption was assessed using Schoenfeld residuals. Inverse probability of treatment weighting was used to generate covariate-adjusted Kaplan-Meier survival curves stratified by allocation period. Within each allocation period, a propensity score model estimated the probability of being female conditional on baseline covariates. Stabilized weights, trimmed at the 1st and 99th percentiles, were applied to balance covariate distributions between sexes. Differences between adjusted survival curves were tested using the Pepe-Fleming integral-based test. Number-at-risk tables reflect unweighted (raw) patient counts. A covariate-adjusted Kaplan-Meier survival curve stratified by device type in the postallocation period was also generated. All analyses were performed in R (version 4.1.1; 2021-08-10; R Foundation for Statistical Computing).

## Results

### Sex-specific trends in the use of tMCS

**Figure 1:**
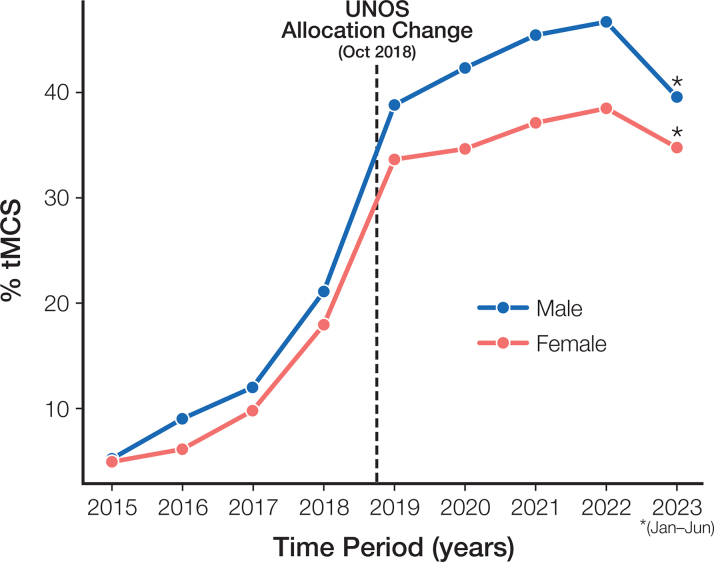
**Sex-Based Trends in Temporary Mechanical Circulatory Support at Listing** This graph depicts trends in the use of temporary mechanical support as bridge to orthotopic heart transplant during the time period from October 2015 to June 2023 stratified by sex. It shows the percentage of men and that of women bridged with devices each year. Dashed line indicates the start of the 2018 United Network for Organ Sharing allocation change policies. tMCS = temporary mechanical circulatory support; UNOS = United Network for Organ Sharing.

**Figure 2:**
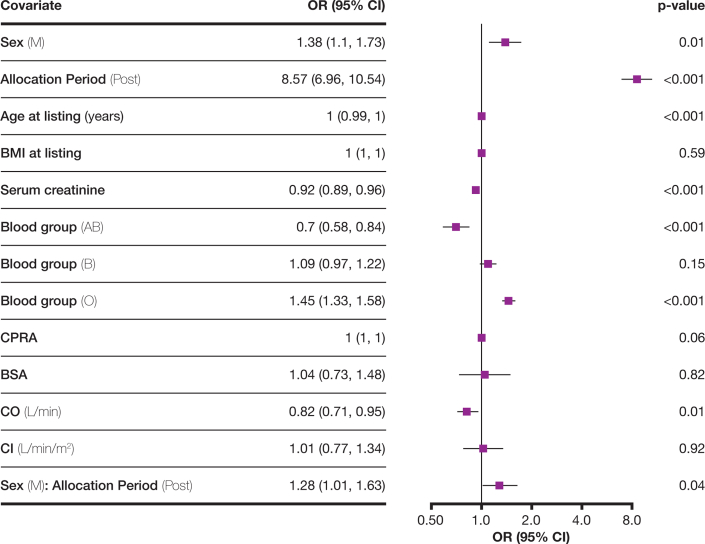
**Predictors of Temporary Mechanical Support at Heart Transplant Listing** Multivariate analysis of factors associated with the use of temporary mechanical support among all patients listed for orthotopic heart transplant between October 2015 and June 2023. For blood group, type A served as the reference. BMI = body mass index; BSA = body surface area; CO = cardiac output; CPRA = calculated panel reactive antibody; CI = cardiac index.

From October 2015 to June 2023 there was a notable surge in the overall use of tMCS as a bridge to OHT. Among women, tMCS use increased from approximately 8.4% to 36% and for men from 11% to 42% (Figure 1). For all-comers listed for OHT, listing during the post allocation period (OR: 8.57 [6.96, 10.54] *P* < 0.001), male sex (OR: 1.38 [1.1,1.73]; *P* = 0.01) and blood type O (OR: 1.45 [1.33,1.58]; *P* < 0.001] were independently associated with the use of tMCS (Figure 2). Women comprised 23% of patients listed for OHT on tMCS, both before and after the allocation change. A higher proportion of women on tMCS underwent OHT compared with men before the allocation change (74% vs 66%; *P* = 0.026); however, postallocation change the frequency of OHT was similar for both women and men on tMCS, 87% and 89%, respectively (Table 1), with overall increased odds of receiving OHT in the post allocation period (OR: 2.07 [1.43, 2.99]; *P* < 0.001) (Supplemental Figure 2).

**Table 1:** Baseline Characteristics and Outcomes for Patients Listed for Orthotopic Heart Transplant on Temporary Mechanical Circulatory Support

	Preallocation Change			Postallocation Change		
	Women (n = 203)	Men (n = 697)	*P* Value	Women (n = 1,852)	Men (n = 6,102)	*P* Value
Transplanted	150 (74%)	458 (66%)	**0.026**	1,618 (87%)	5,411 (89%)	0.12
Age, y	55 (44, 62)	56 (46, 63)	0.17	54 (41, 62)	57 (47, 63)	**<0.001**
BMI	28.0 (24.2, 32.4)	27.7 (24.5, 31.6)	>0.9	26.9 (23.0, 31.3)	27.2 (23.9, 30.9)	**0.005**
Diabetes, %	24	35	**0.005**	25	32	**<0.001**
Implantable defibrillator, %	66	71	0.17	59	65	**<0.001**
Creatinine, mg/dL	0.97 (0.80, 1.26)	1.21 (1.00, 1.55)	**<0.001**	1.00 (0.78, 1.3)	1.20 (0.97, 1.55)	**<0.001**
Blood type			0.23			0.062
A	89 (44%)	259 (37%)		613 (33%)	2,197 (36%)	
AB	10 (4.9%)	26 (3.7%)		67 (3.6%)	255 (4.2%)	
B	27 (13%)	98 (14%)		30 (16%)	946 (16%)	
O	77 (38%)	314 (45%)		869 (47%)	2,704 (44%)	
Cardiac output L/min	3.75 (2.92, 4.57)	4.31 (3.50, 5.23)	**<0.001**	3.40 (2.80, 4.40)	4.00 (3.20, 4.90)	**<0.001**
Body surface area, m^2^	1.84 (1.67, 2.00)	2.08 (1.92, 2.25)	**<0.001**	1.80 (1.65, 1.96)	2.06 (1.90, 2.23)	**<0.001**
Cardiac index L/min/m^2^	2.03 (1.64, 2.44)	2.09 (1.74, 2.53)	0.21	1.92 (1.57, 2.39)	1.92 (1.58, 2.38)	0.89
Mean pulmonary artery pressure, mm Hg	28 (22, 36)	29 (22, 36)	0.49	31 (24, 38)	32 (25, 39)	**<0.001**
Pulmonary capillary wedge pressure, mm Hg	19 (12, 25)	20 (13, 26)	0.37	21 (16, 27)	23 (16, 28)	**<0.001**
MCS type			**0.002**			**<0.001**
IABP	9 (4.4%)	15 (2.2%)		1,235 (67%)	3,719 (61%)	
Nondischargeable/percutaneous device	181 (89%)	575 (82%)		360 (19%)	1712 (28%)	
TAH	12 (5.9%)	99 (14%)		11 (0.6%)	52 (0.9%)	
VA ECMO	1 (0.5%)	8 (1.1%)		246 (13%)	619 (10%)	
UNOS Status attained, %						
1A	80	80	0.83	-	-	
1B	56	62	0.14	-	-	
1	-	-	-	21	21	0.96
2	-	-	-	81	84	**0.004**
3	-	-	-	15	16	0.13
Time on Waitlist, Days	76 (21, 192)	84 (29, 205)	0.2	13 (5, 38)	16 (7, 47)	**<0.001**
Died on Waitlist	20 (9.9%)	93 (13%)	0.2	67 (3.6%)	233 (3.8%)	0.7

Values are n (%) or median (IQR). **Bold** indicates *P* values that are statistically significant.

BMI = body mass index; IABP = intra-aortic balloon pump; MCS = mechanical circulatory support; TAH = total artificial heart; UNOS = United Network for Organ Sharing; VA ECMO = venoarterial extracorporeal membrane oxygenation.

Women in the postallocation change period were more likely to be bridged via IABP compared with men (67% vs 61%; *P* < 0.001) and have lower use of nondischargeable MCS (19% vs 28%; *P* < 0.001). The use of ECMO has increased for both men (pre 1.1% vs post 10%) and women (pre 0.5% vs post 13%). On the contrary, the utilization of TAH has markedly decreased since the allocation change for both sexes (men: pre 14% vs post 0.9%; women: pre 5.9% vs post 0.6%). The majority of men and women listed on tMCS post allocation change were listed status 2, although women less so than men (81% vs 84%; *P* = 0.004).

### Sex-related clinical differences

Baseline characteristics of patients at time of listing are illustrated in Table 1. Postallocation change, women on tMCS were younger (54 vs 57 years; *P* < 0.001), with lower BMI (26.9 vs 27.2; *P* = 0.006) and less likely to have an implantable defibrillator (59% vs 65%; *P* < 0.001) compared with men. In both the preallocation and post-allocation periods, women were less likely to be diabetic. Before the allocation change, filling pressures including mean pulmonary artery pressure (PAPm) and pulmonary capillary wedge pressure (PCWP) were similar between the sexes. However, the postallocation change era filling pressures including PAPm (31 mm Hg vs 32 mm Hg *P* < 0.001) and PCWP (21 mm Hg vs 23 mm Hg *P* < 0.001) were lower in women at the time of listing. Women had lower CO at time of listing preallocation (3.75 vs 4.31 L/min *P* < 0.001) and postallocation (3.4 vs 4 L/min *P* < 0.001) change, although with similar CI (pre: 2.03 vs 2.09 L/min m^2^ *P* = 0.21, post: 1.92 vs 1.92 L/min m^2^ *P* = 0.89).

### Trends in outcomes

**Figure 3:**
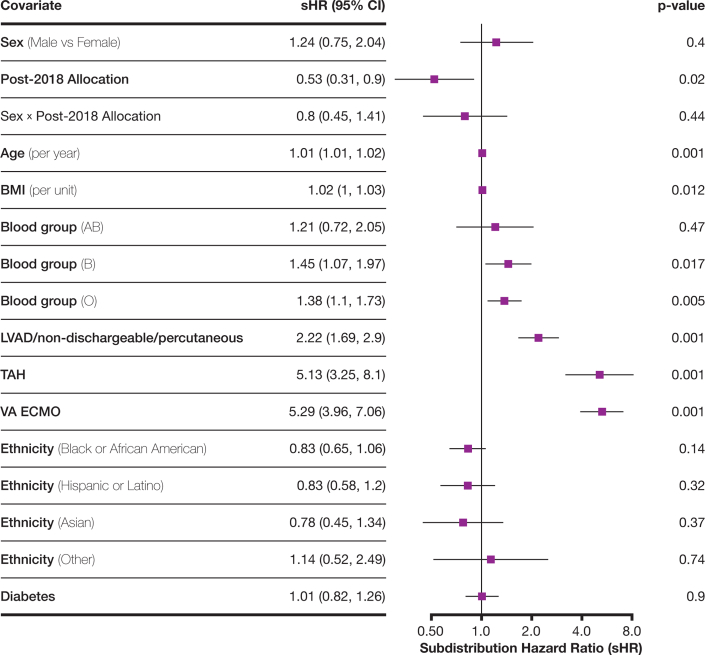
**Predictors of Waitlist Mortality** Factors associated with waitlist survival for patients on temporary mechanical circulatory support listed for orthotopic heart transplant. For Blood Group, type A served as the reference. For devices, the intra-aortic balloon pump (IABP) served as the reference. LVAD = left ventricular assist device; TAH = total artificial heart; VA ECMO = venoarterial extracorporeal membrane oxygenation; other abbreviation as in Figure 2.

Time on the waitlist has decreased for both men (pre 84 vs post 16 days) and women (pre 76 vs post 13 days) from preallocation to postallocation change when bridged with tMCS. Women, however, have shorter waitlist times compared with men postallocation change (13 days vs 16 days; *P* < 0.001). Death on the waitlist decreased for both sexes (men: pre 9.9% vs post 3.6%, women: pre 13% vs post 3.8%) (Table 1). The competing risks model identified several predictors of waitlist mortality (Figure 3). Patients listed after the allocation change had lower subdistribution hazard of waitlist death (subdistribution HR [sHR]: 0.44; 95% CI: 0.33-0.57; *P* < 0.001), consistent with observed improvements in waitlist times for both sexes. Increasing age (sHR: 1.02; 95% CI: 1.01-1.02; *P* < 0.001) and BMI (sHR: 1.02; 95% CI: 1.00-1.03; *P* = 0.013) were associated with modestly increased subdistribution hazard of waitlist death. Blood type B (sHR: 1.46; 95% CI: 1.07-1.98; *P* = 0.016) or type O (sHR: 1.39; 95% CI: 1.10-1.74; *P* = 0.005) was associated with higher subdistribution hazard of waitlist death compared to type A. The strongest predictor of waitlist death was device type with patients supported with ECMO (sHR: 5.29; 95% CI: 3.98-7.07; *P* < 0.001) or TAH (sHR: 5.18, 95% CI: 3.28-8.18; *P* < 0.001) having markedly higher subdistribution hazard compared to those with IABP. Nondischargeable or percutaneous LVADs (sHR: 2.2; 95% CI: 1.69-2.88; *P* < 0.001) were also associated with increased subdistribution hazard.

**Figure 4:**
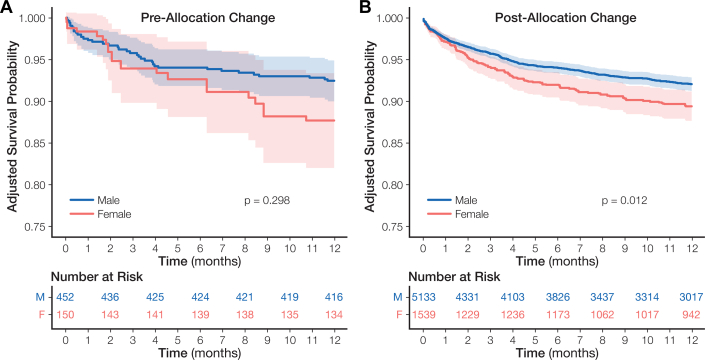
**1-Year Post-Transplant Survival by Sex Across Allocation Eras** Kaplan-Meier Curves showing 1-year post-transplant survival before and after the allocation change stratified by sex. (A) Preallocation period (B) Postallocation period. Inverse probability of treatment weighting method was used to balance baseline characteristics. Covariates include age, ethnicity, body mass index at listing, diabetes status, presence of defibrillator, serum creatinine at the time of transplant, blood type, and device type.

**Figure 5:**
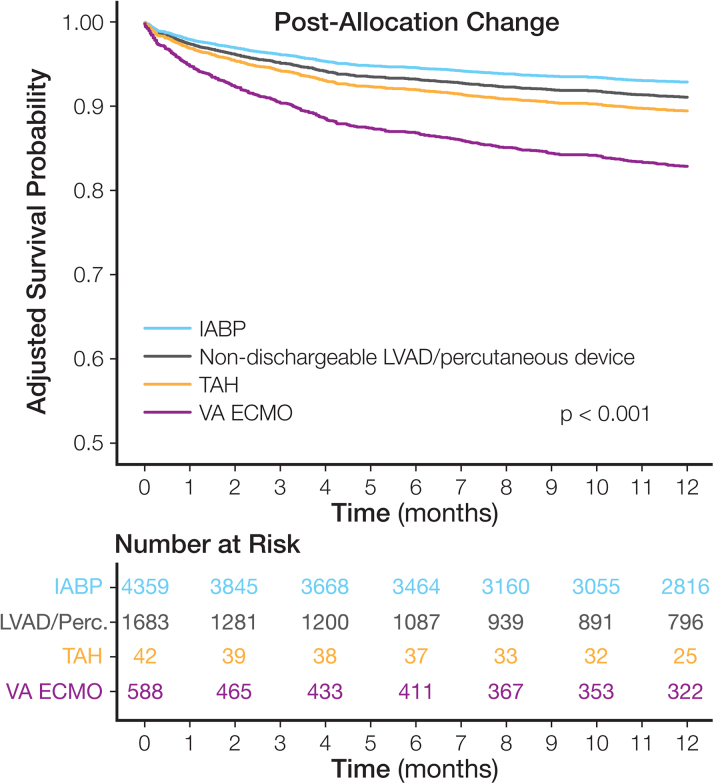
**1-Year Post-Transplant Survival Following the Allocation Change by Device** IABP = intra-aortic balloon pump; other abbreviations as in Figure 3.

The 1-year post-transplant survival was similar between the sexes preallocation change (*P* = 0.298). However, postallocation change, 1-year posttransplant survival was worse for women (*P* = 0.012) (Figure 4). Notably, when stratified by device type, ECMO bridge was associated with an increased risk of death in the new era (*P* < 0.001) (Figure 5).

## Discussion

To our knowledge, this is the largest study to date examining sex specific trends and sex-related differences in the use and outcomes for patients placed on tMCS as bridge to OHT before and after the UNOS allocation change. Our major findings are as follows: 1) patients in the postallocation era, those of male sex, and blood type O have increased likelihood of being placed on tMCS; 2) women account for less than a quarter of those listed on tMCS and are more likely bridged with IABP compared with men; 3) waitlist outcomes improved postallocation change for patients bridged with tMCS independent of sex; however, women experienced worse 1 year post-transplant survival compared to men; and 4) device type is strongly associated with outcomes in those bridged with tMCS to OHT (Central Illustration). Taken together our findings suggest that tMCS, although more frequently used since the allocation change, is more likely to be used in men as a bridge to OHT, with possibly unrealized potential in women, particularly with respect to device selection. The finding of increased tMCS use in patients with blood type O, may be suggestive of decisions being driven by anticipated longer wait times in men who are generally bigger in size, evidenced by higher BSA compared with women in this study (preallocation 1.84 vs 2.08 *P* < 0.001; postallocation 1.8 vs 2.06 *P* < 0.001). However, potential bias for underutilization for heart failure therapies, particularly device therapies, in women have been previously reported. For instance, as demonstrated in prior studies, women in our cohort were less likely to have a defibrillator at the time of listing compared with men.^8^, ^9^, ^10^ Our findings are also consistent with prior studies showing the use of tMCS as well as durable LVAD are less often used in women, at times with resultant worse outcomes.^4^, ^5^, ^6^, ^7^ Vallabhajosyula et al^6^ for example, found that in a cohort of more than 370,000 patients admitted with cardiogenic shock in the setting of acute myocardial infarction, men were significantly more likely to receive tMCS compared with women (50.4% vs 39.5% *P* < 0.001), with higher inpatient mortality, palliative care consultation, and do not resuscitate status observed in women.

**Central Illustration:**
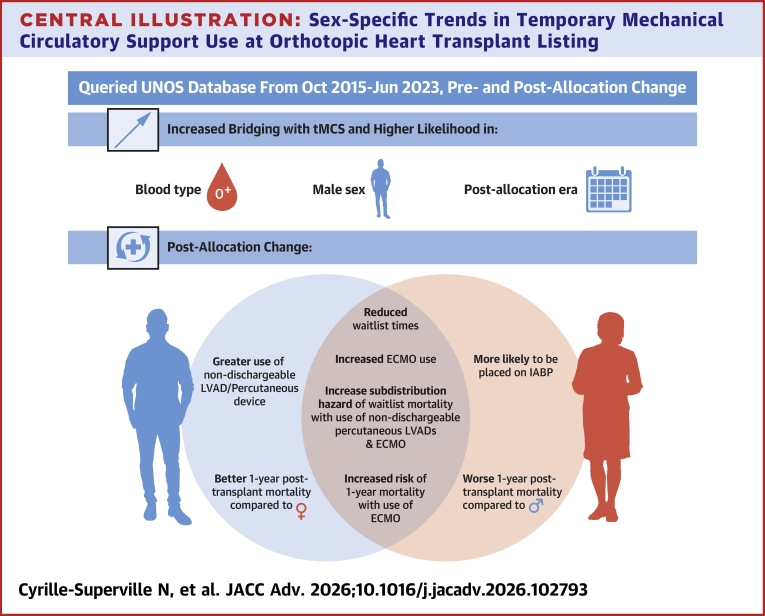
**Sex-Specific Trends in Temporary Mechanical Circulatory Support Use at Orthotopic Heart Transplant Listing** Analysis of the United Network for Organ Sharing database (October 2015 to June 2023) evaluated sex-based trends in the use of temporary mechanical circulatory support (MCS) as bridge to orthotopic heart transplant before and after the 2018 allocation policy changes. Temporary MCS use increased for both sexes in the post-allocation era, though men had greater likelihood of being placed on device support overall and women were more likely placed on intra-aortic balloon pumps as bridge. Extracorporeal membrane oxygenation use increased for both sexes. Waitlist outcomes improved independent of sex, however 1-year post transplant survival was lower for women. Device type was an important determinant of waitlist and post-transplant outcomes. ECMO = extracorporeal membrane oxygenation; IABP = intra-aortic balloon pump; LVAD = left ventricular assist device; OHT = orthotopic heart transplant; TAH = total artificial heart; tMCS = temporary mechanical circulatory support; UNOS = United Network for Organ Sharing.

UNOS data did not provide adequate fidelity to understand what patient features could have affected tMCS bridge strategies. However, women are generally smaller in size as noted previously and so it is possible smaller left ventricular cavities and vasculature could have impacted the decision to proceed with tMCS or the type of tMCS utilized due to concerns for vascular complications.^11^^,^^12^ Furthermore, women are also more likely to be sensitized with preformed anti-human leukocyte antigen antibodies related to prior pregnancies, which could have limited tMCS options due to concern for bleeding and transfusion needs.^13^^,^^14^ Indeed, in this cohort, the limited PRA information available did show higher calculated PRA among women than men (Supplemental Table 2). Hence, the decision to bridge with tMCS may have been weighed against potential bleeding complications that may necessitate blood transfusions, as well as the risk of infectious complications if highly allosensitized individuals undergo desensitization therapies.^14^^,^^15^ Notably, tMCS may help reduce elevated pulmonary pressures, in patients listed for OHT thereby lowering the risk of graft dysfunction and all-cause mortality.^16^^,^^17^ In the postallocation change era, women were found to have statistically significant lower pulmonary filling pressures than men in this cohort (PAPm 31 mm Hg vs 32 mm Hg *P* < 0.001). However, this small difference is unlikely to be clinically meaningful, as both groups exhibited markedly elevated pressures. Furthermore current UNOS hemodynamic criteria for tMCS placement include a PCWP >15 mm Hg, a threshold exceeded by both sexes (PCWP 21 mm Hg in women vs 23 mm Hg in men, *P* < 0.001). Thus, although sex-based statistical differences were observed in filling pressures, they seem unlikely to have influenced clinical decision-making regarding tMCS use in one sex vs the other. It must be noted that, despite these highlighted potential concerns or considerations, the use of tMCS should still be considered for women meeting appropriate criteria, particularly since good outcomes can still be achieved as evidenced by a reduction in waitlist time and improved waitlist mortality in this cohort.

The IABP is the most frequently used device in both men and women listed for OHT on tMCS, with even greater use in women. This may reflect its lower profile and smaller bore design which may be more accommodating to smaller caliber vasculature in women as noted above. It is readily accessible, with relatively fewer complications compared with ECMO or axial flow devices,^18^, ^19^, ^20^, ^21^, ^22^ which may account for its lower subdistribution hazard of waitlist death in this cohort. Nevertheless, as centers gain more experience with axillary devices, such as the Impella 5.5, which provide greater CO augmentation and allow for more patient mobility compared to the femoral IABP, practice patterns could change in the future with greater consideration in women. Trials such as DanGer Shock trial^23^ which demonstrated a reduction in 6-month mortality with use of Impella CP among patients presenting with acute myocardial infarction shock may also serve as an impetus for future changes. Interestingly, despite ECMO bridge to OHT demonstrating continued association with worse outcomes,^20^, ^21^, ^22^ its use has increased among both sexes postallocation change, with a 10-fold increase among men and approximate 25-fold increase among women. Potential attributable factors may center around institutional experience and higher listing status afforded to patients.

In general, the higher listing status granted by tMCS has likely contributed to reduced waitlist time for both sexes, although this is more evident in women given lower BMI/BSA. Consequently, unlike other studies that have shown increased waitlist mortality in women,^5^^,^^24^ in our cohort, the waitlist mortality for men and women is similar and has improved postallocation presumably due to shorter wait periods and increased provider experience leading to fewer device related complications. Given the higher listing status afforded by tMCS in the postallocation change era, compared with the historical reliance on clinical acuity to guide tMCS use in the preallocation change period, one might assume that patients receiving tMCS before the policy change were potentially sicker, thereby contributing to poorer waitlist outcomes. However, the available hemodynamic data did not support this assumption. Patients in the preallocation change era demonstrated lower PAPm and PCWP and a higher CI compared with those in the postallocation era (Table 1), findings that are not consistent with greater illness severity in the earlier cohort. The strong association between device type and waitlist death underscores the increased vulnerability of patients bridged with devices other than IABP and highlights the importance of timely OHT access in this population.

The sex disparity observed in 1 year post-transplant survival in the postallocation era may be related to the type of device used in men vs women. Notably, before the allocation change, both sexes were more likely to be placed on nondischargeable percutaneous/LVADs (women 89%, men 82%) with greater use of TAH (5.9% vs 14%) and ECMO (0.5% vs 1.1%) in men. In the postallocation era, however, despite women having markedly elevated filling pressures (PAPm 31 mm Hg and PCWP 21 mm Hg) and similar CI to men (1.92 vs 1.92 L/min m^2^ *P* = 0.89) they were more likely to be placed on IABP as opposed to nondischargeable percutaneous devices, presumably Impellas, which provide greater augmentation of CO. Consequently, women may have been relatively under supported while awaiting OHT contributing to their post-transplant vulnerability. The greater reduction in waitlist time in women may have partially mitigated the negative impact of being under supported while on wait list. Also of note is the striking 25-fold increase in ECMO use in women. Given their smaller vasculature and susceptibility to vascular and bleeding complications, this escalation may have resulted in a disproportionate risk compared to men, who also experienced increase in ECMO use but to a lesser extent (10-fold increase).

### Study limitations

This study has several limitations inherent in its retrospective design. Clinical information and outcomes were limited by the data available in the UNOS registry. For instance, absolute contraindications to tMCS, including anatomic considerations such as vessel size, are not available in UNOS resulting in potential selection bias. Hence, caution must be exercised when interpreting whether women have underutilization of tMCS, as there may have been objective absolute contraindications for tMCS that are not captured. Future studies could incorporate review of exception requests granted for absolute contraindications as a proxy for such limitations, although such work would require extensive manual adjudication. Antibody sensitization data were limited with high missing rate, hence limiting analysis and conclusions regarding tMCS decision-making based on allosensitization.

Potential information bias and residual confounding are also noteworthy. Provider and center-level variability in candidate selection, approach to listing, waitlist management, and tMCS use, including rate of adoption of various tMCS technologies, could have impacted decisions for tMCS use and outcomes. These practice variations are not comprehensively captured in the UNOS registry and therefore cannot be incorporated into our analyses. Similarly, device-related complications are not detailed in the registry. Organ offers and availability, which vary by geographic region and donor–recipient distance, may affect waitlist times but cannot be fully assessed using UNOS data. Finally, donor-recipient matching considerations—including size, immunologic compatibility, and donor quality—may have influenced post-transplant outcomes and likewise could not be fully accounted for in this analysis.

## Conclusions

There has been a significant increase in the use of tMCS as a bridge to OHT, since the UNOS allocation change, with increased likelihood of devices in men and blood type O patients, presumably due to anticipated longer wait times. Women are less likely to be listed on tMCS and more often on IABP. Although waitlist mortality has improved for both sexes, women experience worse 1-year post transplant survival post allocation change. Device type was found to be the strongest predictor of both waitlist and post-transplant outcomes.

Perspectives **COMPETENCY IN PATIENT CARE AND PROCEDURAL SKILLS:** The use of tMCS as a bridge to OHT has risen, particularly among men and blood type O patients. Improvement in waitlist outcomes for both sexes, suggest potential for broader future use in women. However, the type of device remains critically important given worse 1-year post transplant survival in women.

**TRANSLATIONAL OUTLOOK:** Careful selection of lower profile devices that minimize complications yet provide adequate CO augmentation may help mitigate post-transplant mortality disparities in women. Risk patterns may evolve as centers gain more experience with non-IABP devices influencing future patient outcomes and perhaps even prompting the realignment of how patients are prioritized within the current listing status frameworks.

## Funding support and author disclosures

Dr Cyrille-Superville has received research funding through her institution from the 10.13039/100000002NIH and provides consulting services for and/or receive honoraria from 10.13039/100006400Alnylam, 10.13039/100004319Pfizer and 10.13039/100032703Bridgebio. Dr DeVore has received research funding through his institution from 10.13039/100032830Biofourmis, 10.13039/100032492Bodyport, 10.13039/100014941Cytokinetics, 10.13039/100016473American Regent, Inc, the 10.13039/100000002NIH and 10.13039/100000050NHLBI, 10.13039/100020295Natera, 10.13039/100004336Novartis, Story Health, and Ventricle Health; and provides consulting services for and/or receives honoraria from 10.13039/100020297Abiomed, 10.13039/100032492Bodyport, Cardionomic, LivaNova, Myovant, Natera, NovoNordisk, and 10.13039/100015345Zoll. Dr Patel provides consulting services for Kestra Medical Technologies. Dr Gulati provides consulting services and receives honoraria from 10.13039/100000046Abbott. Dr Ilonze is supported by the Robert A. Winn Excellence in Clinical Trials Career Development Award; and has received consulting fees and honorarium from 10.13039/100032703Bridgebio, 10.13039/100004325Astrazeneca and 10.13039/100004326Bayer. All other authors have reported that they have no relationships relevant to the contents of this paper to disclose.

## References

[1] Bernhardt A.M. (2019). The new tiered allocation system for heart transplantation in the United States: a Faustian bargain. J Heart Lung Transplant.

[2] Shin M., Iyengar A., Helmers M.R. (2022). Modern outcomes of heart-lung transplantation: assessing the impact of the updated United States allocation system. Eur J Cardiothorac Surg.

[3] Cogswell R., John R., Estep J.D. (2020). An early investigation of outcomes with the new 2018 donor heart allocation system in the United States. J Heart Lung Transplant.

[4] Joshi A.A., Lerman J.B., Sajja A.P. (2019). Sex-based differences in left ventricular assist device utilization: insights from the Nationwide Inpatient Sample 2004 to 2016. Circ Heart Fail.

[5] DeFilippis E.M., Truby L.K., Garan A.R. (2019). Sex-related differences in use and outcomes of left ventricular assist devices as bridge to transplantation. JACC Heart Fail.

[6] Vallabhajosyula S., Dunlay S.M., Barsness G.W. (2020). Sex disparities in the use and outcomes of temporary mechanical circulatory support for acute myocardial infarction–cardiogenic shock. CJC Open.

[7] Bardwaj A., Rajapreyer I., Kumar S. (2023). Sex disparities in the use of temporary mechanical circulatory support for nonischemic cardiogenic shock. J Heart Lung Transplant.

[8] Curtis L.H., Al-Khatib S.M., Shea A.M., Hammill B.G., Hernandez A.F., Schulman K.A. (2007). Sex differences in the use of implantable cardioverter-defibrillators for primary and secondary prevention of sudden cardiac death. JAMA.

[9] Gauri A.J., Davis A., Hong T., Burke M.C., Knight B.P. (2006). Disparities in the use of primary prevention and defibrillator therapy among Blacks and women. Am J Med.

[10] Ignaszewski M.T., Daugherty S.L., Russo A.M. (2019). Implantable cardioverter-defibrillators and cardiac resynchronization therapy in women. Heart Fail Clin.

[11] Magnussen C., Bernhardt A.M., Ojeda F.M. (2018). Gender differences and outcomes in left ventricular assist device support: the European Registry for Patients with Mechanical Circulatory support. J Heart Lung Transplant.

[12] Topkara V.K., O’Neill J.K., Carlisle A., Novak E., Silvestry S.C., Ewald G.A. (2014). HeartWare and HeartMate II left ventricular assist devices as bridge to transplantation: a comparative analysis. Ann Thorac Surg.

[13] Hasan A., Kittleson M.M. (2019). Heart transplantation in women. Heart Fail Clin.

[14] Kidambi S., Mohamedali B., Bhat G. (2015). Clinical outcomes in sensitized heart transplant patients bridged with ventricular assist devices. Clin Transplant.

[15] Urban M., Gazdic T., Slimackova E. (2012). Alloimmunosensitization in left ventricular assist device recipients and impact on post-transplantation outcome. ASAIO J.

[16] Jedeon Z., Baker W., Pillai A. (2026). Effect of temporary mechanical circulatory support on elevated pulmonary pressures in patients awaiting heart transplantation. J Card Fail.

[17] Karki D., Chaudhary A., Bhusal S. (2025). Impact of pretransplant mechanical circulatory support on pulmonary artery pressure in heart transplant recipients: a retrospective cohort study. Chest.

[18] Fukuhara S., Takeda K., Kurlansky P.A., Naka Y., Takayama H. (2018). Extracorporeal membrane oxygenation as a direct bridge to heart transplantation in adults. J Thorac Cardiovasc Surg.

[19] Barge-Caballero E., Almenar-Bonet L., Gonzalez-Vilchez F. (2018). Clinical outcomes of temporary mechanical circulatory support as a direct bridge to heart transplantation: a nationwide Spanish registry. Eur J Heart Fail.

[20] Poptsov V., Spirina E., Dogonasheva A., Zolotova E. (2019). Five years’ experience with peripheral venoarterial ECMO for mechanical bridge to heart transplantation. J Thorac Dis.

[21] Lui C., Fraser C.D., Suarez-Pierre A. (2021). Evaluation of extracorporeal membrane oxygenation therapy as a bridging method. Ann Thorac Surg.

[22] Ali J.M., Abu-Omar Y. (2020). Complications associated with mechanical circulatory support. Ann Transl Med.

[23] Møller J., Engstrøm T., Jensen L.O. (2024). Microaxial flow pump or standard care in infarct-related cardiogenic shock. N Engl J Med.

[24] Hsich E.M., Blackstone E.H., Thuita L. (2017). Sex differences in mortality based on United Network for Organ Sharing status while awaiting heart transplantation. Circ Heart Fail.

