# Supplementary material for: Sex-Specific Trends in Temporary Mechanical Circulatory Support Use as Bridge to Orthotopic Heart Transplant
Source: JACC Adv. 2026 Jun 17;5(6):102793. doi: 10.1016/j.jacadv.2026.102793 (PMC13309315; doi:10.1016/j.jacadv.2026.102793)
Supplement: Supplemental Material [file mmc1.pdf]

Supplemental Appendix Table of Contents

Title	Page
Figure Legends	2
Figures	3-5
Tables	6-7
Strobe Check List	8-9

Figure Legends

Supplemental Figure 1. Directed Acyclic Graph showing Probable Causal Relationships Between Variables. This depicts unidirectional relationships based on prior literature and subject-matter knowledge which aided in the selection of covariates for adjustment and helped identify potential sources of confounding, mediation, and bias.

Supplemental Figure 2. Factors Associated with Receiving a Heart Transplant. Forest plot showing multivariate analysis of factors associated with receiving a heart transplant for all patients listed on temporary mechanical support from October 2015 to June 2023. For Blood Group, type A is the reference. For devices, the intra-aortic balloon pump (IABP) is the reference. BMI = Body Mass Index, IABP = Intra-aortic balloon pump, MCS = Mechanical Circulatory Support, TAH = Total Artificial Heart, VAD = Ventricular Assist Device, VA ECMO= Veno-arterial extracorporeal membrane oxygenation

Supplemental Figure 3: Factors Associated with One-year post Transplant Survival. Forest plot showing multivariate analysis of factors associated with one-year post-transplant survival. For Blood Group, type A is the reference. For devices, the intra-aortic balloon pump (IABP) is the reference. BMI = Body Mass Index, IABP = Intra-aortic balloon pump, MCS = Mechanical Circulatory Support, TAH = Total Artificial Heart, VAD = Ventricular Assist Device, VA ECMO= Veno-arterial extracorporeal membrane oxygenation

Supplemental Figure 1. Directed Acyclic Graph showing Probable Causal Relationships Between Variables.

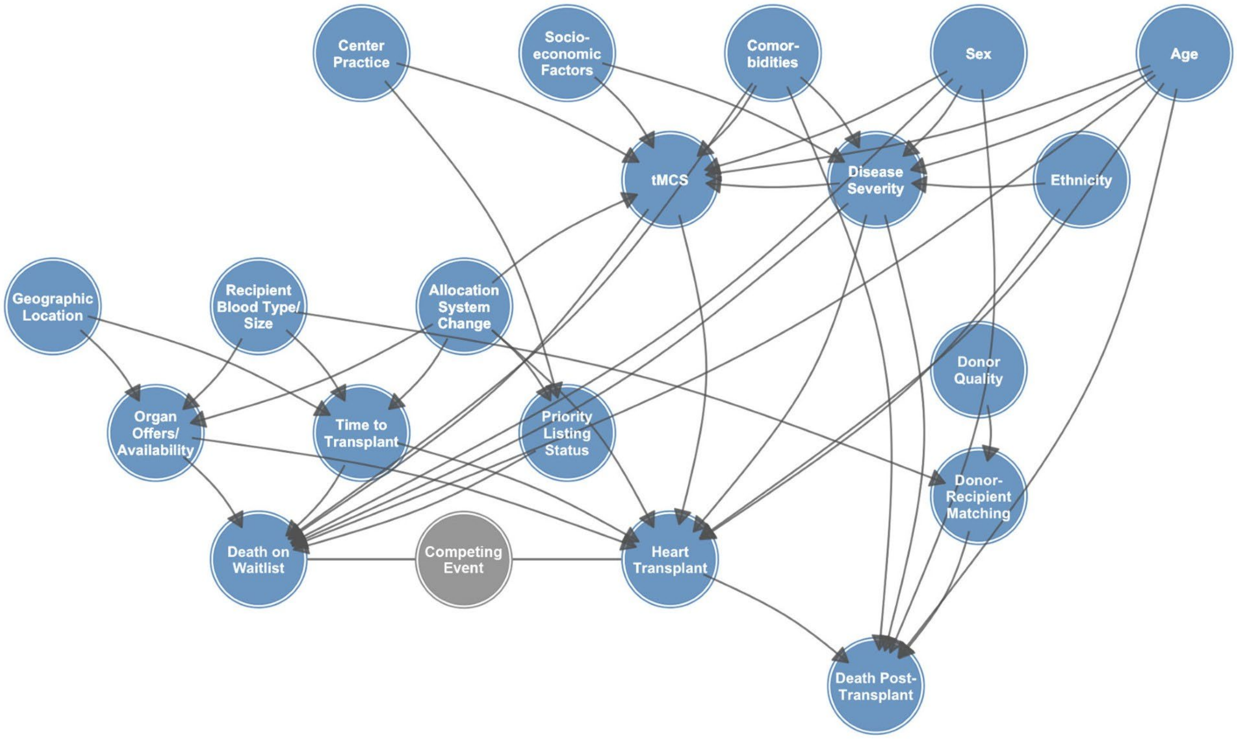

Supplemental Figure 2. Factors Associated with Receiving a Heart Transplant.

Covariate	OR (95% CI)	p-value
**Sex** (M)	0.75 (0.52, 1.07)	0.11
**Allocation Period** (Post)	2.07 (1.43, 2.99)	<0.001
**Age at listing** (years)	0.99 (0.98, 0.99)	<0.001
**Ethnicity** (Black or African American)	1.04 (0.89, 1.22)	0.62
**Ethnicity** (Hispanic or Latino)	1.02 (0.82, 1.27)	0.87
**Ethnicity** (Asian)	0.95 (0.69, 1.31)	0.74
**Ethnicity** (Other)	0.95 (0.56, 1.61)	0.84
**BMI at listing**	0.98 (0.97, 0.99)	<0.001
**Diabetes**	0.93 (0.8, 1.07)	0.28
**Blood group** (AB)	1.15 (0.79, 1.66)	0.47
**Blood group** (B)	0.79 (0.65, 0.97)	0.02
**Blood group** (O)	0.73 (0.63, 0.85)	<0.001
**Implantable defibrillator at time of transplant**	0.94 (0.81, 1.08)	0.37
**MCS Device Type** (VAD/Non-dischargeable VAD/Percutaneous device)	0.57 (0.48, 0.67)	<0.001
**MCS Device Type** (TAH)	0.33 (0.23, 0.48)	<0.001
**MCS Device Type** (VA ECMO)	0.21 (0.17, 0.25)	<0.001
**Sex** (M)**: Allocation Period** (Post)	1.53 (1.03, 2.27)	0.04

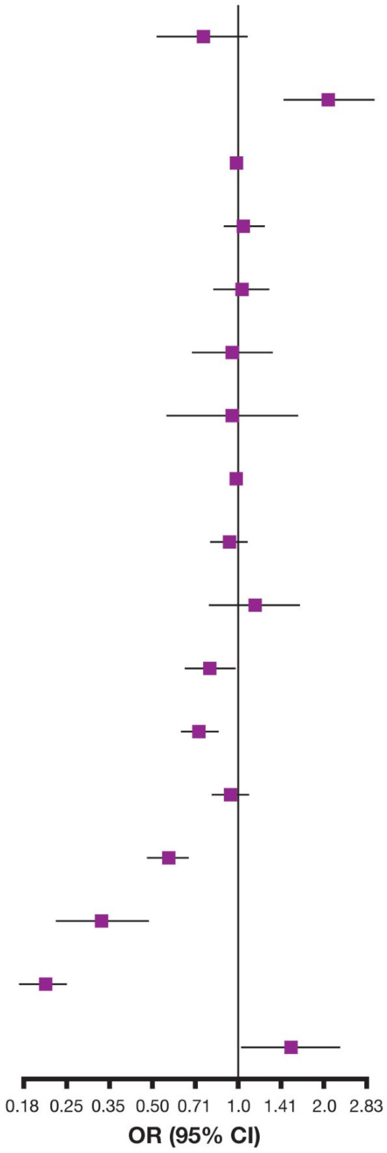

Supplemental Figure 3: Factors Associated with One-year post Transplant Survival

Covariate	HR (95% CI)	p-value
**Sex** (M)	0.69 (0.38, 1.24)	0.21
**Allocation Period** (Post)	1.03 (0.59, 1.77)	0.93
**Age at listing** (years)	1.02 (1.01, 1.03)	<0.001
**Ethnicity** (Black or African American)	0.92 (0.75, 1.14)	0.46
**Ethnicity** (Hispanic or Latino)	1.01 (0.75, 1.36)	0.94
**Ethnicity** (Asian)	1.66 (1.14, 2.42)	0.01
**Ethnicity** (Other)	0.57 (0.21, 1.52)	0.26
**BMI at listing**	1.04 (1.02, 1.05)	<0.001
**Serum creatinine at time of transplant**	1.14 (1.07, 1.22)	<0.001
**Diabetes**	1.02 (0.85, 1.23)	0.81
**Blood group** (AB)	0.98 (0.64, 1.52)	0.94
**Blood group** (B)	0.82 (0.62, 1.09)	0.17
**Blood group** (O)	1.1 (0.91, 1.33)	0.34
**Implantable defibrillator at time of transplant**	1.02 (0.85, 1.23)	0.82
**MCS Device Type** (VAD/Non-dischargeable VAD/Percutaneous device)	1.24 (1, 1.54)	0.05
**MCS Device Type** (TAH)	2.44 (1.39, 4.3)	<0.001
**MCS Device Type** (VA ECMO)	2.5 (1.92, 3.24)	<0.001
**Sex** (M)**: Allocation Period** (Post)	1.11 (0.59, 2.07)	0.74

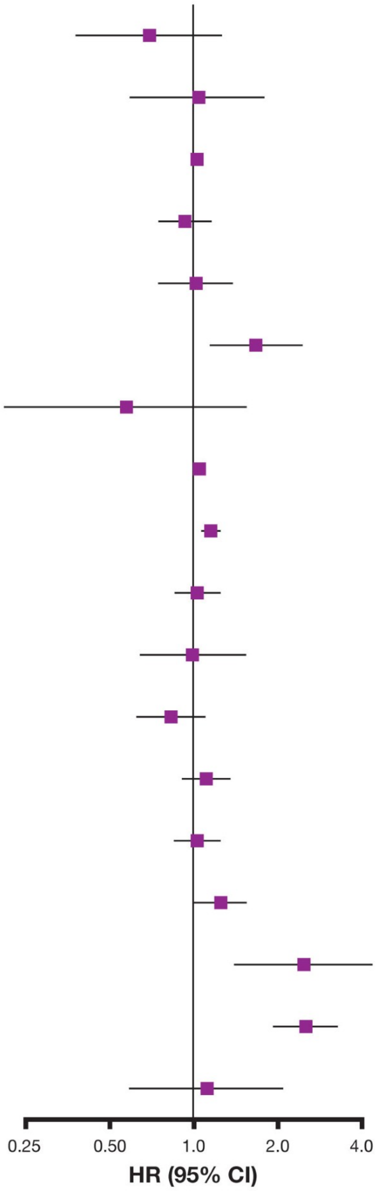

Tables

Supplemental Table 1. Proportion of Patients on Temporary Mechanical Support at the Time of Transplant Listing

	Pre-Allocation			Post-Allocation		
**Characteristics**	**F N =203**	**M N=697**	**p-value**	**F N =1,852**	**M N =6,102**	**p-value**
**Was patient put on tMCS>7 days after listing? n (%)**			0.76			0.28
**No**	141 (69)	492 (71)		1,368 (74)	4,429 (73)	
**Yes**	62 (31)	205 (29)		484 (26)	1,673 (27)	

F= Female, M = Male, MCS = Mechanical Circulatory Support

Supplemental Table 2. Calculated Panel Reactive Antibody for Patients Bridged with Temporary Support to Orthotopic Heart Transplant

	**Pre-Allocation Change**			**Post Allocation Change**		
Characteristic	**Women n=203**	**Men n=697**	**p-value**	**Women n=1852**	**Men n=6102**	**p-value**
Most recent CPRA	18 (32)	7 (16)	**0.002**	20 (31)	7 (17)	**<0.001**
Unknown	80	328		705	2205	
Peak CPRA	23 (36)	11 (21)	**0.004**	24 (33)	10 (20)	**<0.001**
Unknown	80	327		705	2209	

Values are expressed as mean (SD) as appropriate. **Bold** indicates p-values that are statistically significant
CPRA = Calculated Panel Reactive Antibody

Strobe Check List

STROBE Checklist – Retrospective Cohort Study

Checklist for transparent reporting of a retrospective cohort study. Click or mark checkboxes as completed.

1. Title & Abstract

Item	✓/□
Indicate retrospective cohort design in title or abstract.	✓ Indicated in Abstract
Provide a balanced abstract summarizing methods and findings.	✓

2. Introduction

Item	✓/□
Explain the scientific background and rationale.	✓
State objectives and hypotheses.	✓

3. Methods

Item	✓/□
Describe study design explicitly as retrospective cohort.	✓
Explain setting, location, and relevant dates (exposure, follow-up, data retrieval).	✓
Define eligibility criteria and case identification methods.	✓
Describe exposures, outcomes, confounders, and diagnostic criteria.	✓
Provide data sources and measurement details.	✓
Discuss efforts to address bias.	✓
Explain study size determination.	✓
Describe handling of quantitative variables.	✓
Describe all statistical methods including confounder control.	✓
Explain handling of missing data.	✓
Describe subgroup, interaction or sensitivity analyses.	✓

4. Results

Item	✓/☐
Report numbers of individuals at each stage (screened, eligible, included).	✓
Provide reasons for non-participation at each stage.	✓
Describe participant characteristics and missing data.	✓
Report outcome events and summary measures.	✓
Present crude and adjusted estimates with confidence intervals.	✓
Report subgroup or sensitivity analyses.	✓

5. Discussion

Item	✓/☐
Summarize key results in relation to study objectives.	✓
Discuss limitations including sources of bias and imprecision.	✓
Consider direction and magnitude of potential bias.	✓
Provide a cautious interpretation considering other evidence.	✓
Discuss generalisability of the results.	✓

6. Other Information

Item	✓/☐
Provide funding sources and roles.	✓
State IRB approval or exemption.	✓
